# From posts to engagement: comparing TikTok and Instagram communication among Czech sports teams

**DOI:** 10.3389/fspor.2026.1964766

**Published:** 2026-10-08

**Authors:** Kateřina Bočková, Imran Ahmed Shahzad, David Anthony Procházka

**Affiliations:** 1 Department of Management and Economics, DTI University, Dubnica nad Váhom, Slovakia; 2 School of Business and IT, University of New York in Prague, Prague, Czechia

**Keywords:** anova, Czech republic, fan engagement, instagram, social media, sports marketing, TikTok

## Abstract

**Introduction:**

Social media platforms have become important communication channels for sports organisations, yet audience engagement may vary according to platform, content format, publication day, and posting time. This study aimed to compare engagement performance on TikTok and Instagram among selected Czech sports teams and to identify platform-, content-, day-, and time-specific patterns associated with audience interaction.

**Methods:**

The study employed a quantitative content analysis of 7,721 social media content items published by 15 selected Czech sports organisations in 2024. Data were obtained through Popsters and c using Engagement Rate (ER), Love Rate (LR), and Talk Rate (TR). In addition, a selected subset of 115 content items was examined to identify content-specific engagement patterns across TikTok videos, Instagram Reels, and standard Instagram posts. Differences related to platform, publication day, and posting time were assessed using descriptive and inferential statistical analyses.

**Results:**

Descriptive results showed higher engagement values for TikTok across several metrics; however, inferential analysis did not identify a statistically significant difference in engagement between the platforms. Content analysis of the selected subset indicated that Player/Athlete Persona and Fan Engagement were prominently represented among TikTok videos and Instagram Reels, whereas Current Event and Match Highlights, Results or Previews were more strongly represented among standard Instagram posts. No statistically significant differences in engagement were identified according to publication day or posting time.

**Discussion:**

The findings indicate platform-, format-, and content-specific descriptive engagement patterns rather than statistically confirmed superiority of either TikTok or Instagram. The results suggest that Czech sports organisations may benefit from adapting organic social media communication to the specific engagement characteristics of individual platforms and content formats. However, the findings should be interpreted in light of the purposive sample and observational research design, which limit causal inference and broader generalisability.

## 1 Introduction

The landscape of sports marketing has undergone a substantial transformation, shifting from a predominant reliance on traditional media, such as radio, television, and print, towards digitally mediated communication and social media. Social media platforms enable sports organisations to communicate directly with fans, provide real time updates, share “behind the scenes” content, and develop interactive forms of audience engagement. This transformation has also changed the ways in which sports organisations communicate their identities and manage relationships with their audiences. Short-form, visually oriented content has become particularly relevant within this environment, especially among younger audiences whose media consumption increasingly involves on the demand and mobile first formats (1). TikTok and Instagram consequently represent important communication channels through which sports teams can distribute creative, interactive, and fan-oriented content.

TikTok and Instagram nevertheless differ in their platform architecture, content formats, and mechanisms of content distribution. TikTok is strongly associated with short-form video, algorithmically mediated content discovery, and trend-oriented communication, whereas Instagram combines several formats, including conventional posts and Reels, that enable different forms of visual communication and interaction. Previous research has demonstrated the importance of visual content and social media for relationship development and brand management in professional sport (2, 3). Short form social media content can also facilitate interaction between sports organisations, athletes, and their audiences; particularly, when content is adapted to the communication characteristics of the respective platform (4). These platform differences provide a relevant basis for examining whether observable audience engagement varies between TikTok and Instagram and across different types of content.

Despite the growing body of international research on social media in sport, the Czech and broader Central European context remains comparatively underexplored. Existing research has established the importance of digital communication and audience engagement in sports marketing, but much of the literature has examined international markets, individual athletes, or professional sport environments that may differ substantially from the conditions faced by Czech sports organisations. This distinction is relevant because social media strategies are implemented within specific organisational, economic, and cultural contexts. For sports teams with more limited communication and marketing resources, organic social media may represent an accessible means of maintaining interaction with existing audiences and potentially extending their digital visibility. The Czech setting therefore provides a useful context in which to investigate engagement patterns without assuming that findings from larger international sport markets are directly transferable.

A further research need concerns the comparative assessment of observable engagement across social media platforms. Previous research has employed social media engagement indicators to examine audience responses to digital content, while platform-specific analytics provide opportunities to distinguish different dimensions of interaction (3, 5). In the present study, Engagement Rate (ER), Love Rate (LR), and Talk Rate (TR) are used as indicators of observable audience interaction. These measures enable comparison of overall engagement, liking behaviour, and commenting behaviour across the analysed content. Importantly, they are treated as measures of engagement performance rather than direct measures of brand loyalty, brand development, or overall marketing effectiveness. This distinction is necessary because broader brand-related outcomes require additional behavioural or attitudinal evidence beyond publicly observable social media interactions.

The relevance of such engagement extends beyond individual posts. Social media can contribute to the broader processes through which sports organisations communicate their identities, maintain relationships with fans, and increase their visibility (2). At the same time, engagement outcomes cannot be assumed to result solely from the platform used. Content characteristics, communication strategies, posting practices, and audience responses may all contribute to observed differences. Recent research therefore emphasises the importance of understanding social media engagement as a platform- and content-dependent phenomenon rather than attributing performance automatically to a particular technological environment (3, 4). Similarly, research on TikTok demonstrates the relevance of short-form content for sport-related audience interaction, particularly among younger social media users (4). These findings provide a basis for comparative analysis while avoiding causal assumptions about platform algorithms or audience demographics that cannot be established from post-level observational data.

Against this background, the present study addresses a gap in the empirical understanding of social media engagement among Czech sports teams by comparatively examining TikTok and Instagram using organic, publicly observable social media content. Rather than assessing overall brand-building effectiveness, the study focuses specifically on measurable engagement performance and on descriptive patterns associated with platform, content type, publication day, and posting time. This approach enables the study to contribute evidence from an underrepresented Central European sporting context while also generating practically relevant insights for sports organisations operating with different levels of resources.

Therefore, the primary objective of this study is to compare engagement performance on TikTok and Instagram among selected Czech sports teams and to identify platform-, content-, day-, and time-specific patterns associated with audience interaction. Specifically, the study examines differences in Engagement Rate (ER), Love Rate (LR), and Talk Rate (TR), together with descriptive patterns related to content categories and publication timing. In doing so, the research seeks to identify engagement patterns that may inform platform-specific organic social media strategies without interpreting these metrics as direct evidence of brand loyalty or long-term brand development. This more focused perspective allows the study to distinguish empirically observed engagement outcomes from their broader potential implications for sports marketing and digital brand communication.

## 2 Literature review

Historically, sports marketing relied predominantly on traditional media such as television, newspapers, and radio. Digitalisation has substantially transformed this environment by enabling sports organisations to communicate directly and continuously with their audiences through social media (6). This transformation has increased the strategic importance of digital communication within contemporary sport management and marketing (7, 8). Social media platforms provide sports organisations with opportunities to distribute content, communicate organisational and athlete identities, and encourage observable audience interaction (3). Research has consequently emphasised the role of social media in facilitating real-time communication and interaction between sports organisations and their audiences (6, 9).

Within this changing environment, Instagram and TikTok have become particularly relevant because of their emphasis on visual and short-form content. The growing consumption of short-form content among younger audiences has increased the strategic relevance of these platforms within digital marketing (1, 10). However, Instagram and TikTok differ in their platform architecture, content formats, and mechanisms of content discovery. Instagram integrates conventional posts, Stories, and Reels, whereas TikTok is primarily structured around short-form video and algorithmically mediated content discovery. These characteristics provide sports organisations with different opportunities for presenting content and interacting with audiences (3, 4). More broadly, Koronios (11) identifies social media as an increasingly important component of the sport business environment, connecting digital communication, audience interaction, marketing, and relationship-management processes. Table 1 summarises the principal tools and features of TikTok and Instagram that are relevant to these communication processes.

**Table 1:** Summary of core tools and features of TikTok and Instagram.

Tool/Feature	TikTok	Instagram	Description/Purpose
Short-form videos	Core feature	Reels	Short, visually dynamic video content designed for rapid consumption and audience interaction.
Live streams	Available (TikTok LIVE)	Available (Instagram Live)	Real-time video streaming used for Q&As, behind-the-scenes access, announcements, and live interaction with audiences.
Standard posts	Not available as a core static-post format	Core feature	Feed-based content that may include images, carousels, and videos and remains visible on the profile unless removed.
Stories	Available	Available	Temporary 24 h content used for quick updates, polls, behind-the-scenes communication, and audience interaction.
For You Page/Explore	For You Page (FYP)—core discovery feature	Explore—available	Discovery-oriented areas through which users may encounter content from accounts they do not already follow.
AR filters/effects	Available	Available	Augmented-reality and visual effects that can be applied to photos and videos to enhance creative presentation.
Duets/Remix	Duets	Remix for Reels	Interactive features allowing users to respond to, reinterpret, or create content alongside existing videos.

Source: Researcher.

Platform-specific communication is particularly important in sport because the relationship between organisations, athletes, and fans is highly interactive. TikTok content frequently relies on short, dynamic, participatory, and visually distinctive forms of communication, whereas Instagram combines short-form video with more established visual communication formats. Different visual and communicative structures may consequently encourage different forms of audience response (12, 13). Research focused specifically on sports organisations likewise demonstrates that social networks have become established channels of marketing communication and that their effective use requires content and communication practices adapted to both organisational objectives and platform characteristics (9). Such evidence supports the examination of platform-specific engagement without assuming that the use of a particular platform automatically produces stronger branding outcomes.

Viral marketing represents another relevant dimension of social media communication. Content capable of rapid diffusion can increase visibility and audience exposure, particularly when it generates emotional responses or encourages users to share and interact with it (14, 15). Humour, emotional resonance, and shareability have therefore become important characteristics of digital communication strategies (16). Research has also identified the potential economic and communication value of viral content for sports organisations (17). Nevertheless, virality is inherently difficult to predict and sustain. Campaign momentum can decline rapidly without appropriate follow-up communication (18), while audience responses to digital content remain variable (19). Furthermore, developing effective digital sport marketing content requires strategic planning and adaptation to platform-specific communication practices (9, 17). Viral communication should therefore be understood as one possible component of social media strategy rather than as a guaranteed outcome of using a particular platform. When successful, it may contribute to visibility and audience interaction (20).

The strategic relevance of social media is also evident in contemporary sport sponsorship and event management. Koronios et al. (21) demonstrate that social media can function as an important mediating mechanism connecting sponsorship-related factors with spectator engagement in the context of a major sporting event. Their findings reinforce the broader argument that social media should not be considered merely as a distribution channel but as part of the strategic management of relationships among sports organisations, commercial partners, and audiences. At the same time, engagement in such contexts is multidimensional and should not be reduced automatically to individual post-level metrics. This distinction is particularly important for the present study, which examines observable interactions with social media content rather than attitudes towards brands, purchase intentions, or long-term loyalty.

The measurement of social media engagement therefore represents an important methodological issue. Engagement Rate (ER), Love Rate (LR), and Talk Rate (TR) provide indicators of different dimensions of observable audience interaction with published content (22). Previous research has used engagement-related measures to evaluate responses to digital sport content and to compare communication formats and strategies (3, 4). In the present context, these indicators provide a basis for examining whether patterns of likes, comments, and overall interactions vary across TikTok, Instagram Reels, and Instagram posts. However, such metrics should not be interpreted as direct measures of brand loyalty, brand equity, or overall marketing effectiveness. Research showing relationships between interactive or community-oriented content and broader brand-related outcomes (23–25) provides important theoretical context, but these broader outcomes require measurements beyond the publicly observable engagement indicators used in the present study.

Content characteristics are also central to social media engagement. Sports organisations can publish match information, athlete-focused material, behind-the-scenes content, fan-oriented communication, promotional messages, and entertainment-based posts, among other categories. Different content types may generate different levels and forms of interaction because they fulfil different informational, emotional, and relational functions. This is consistent with research emphasising the importance of athlete-focused and visually engaging social media content for audience interaction (3, 12). Content centred on athletes may be particularly relevant because athletes can serve as identifiable and humanising elements of sports communication, while community-oriented content may facilitate interaction between organisations and fans. These theoretical relationships provide a basis for examining content-specific engagement patterns without assuming that high engagement necessarily demonstrates increased loyalty or long-term brand development.

Posting context and timing represent additional potential sources of variation in audience interaction. Previous research suggests that content associated with live events, topical developments, or periods of greater audience activity may generate different engagement patterns (15, 16). Platform algorithms and content-distribution systems may also influence content visibility, requiring sports organisations to adapt their communication practices as platforms evolve (1, 26). Nevertheless, the influence of these mechanisms should be distinguished from empirically observed post-level engagement. Without direct measures of algorithmic distribution, reach, or individual user behaviour, differences associated with posting day or time should be interpreted as descriptive engagement patterns rather than evidence of specific algorithmic or behavioural mechanisms.

The geographical and organisational context is equally important. Although social media in sport has received substantial international attention, the Czech and wider Central European environment remains less extensively represented in the literature. Earlier Czech research examined the adoption of Web 2.0 tools in sport (27), while subsequent work has highlighted the importance of cultural, demographic, and communication contexts for social media strategies (12, 28). Crossan et al. (29), for example, demonstrate the relevance of brand and internationalisation processes within Czech professional sport, while Švecová (26) provides evidence concerning the digital behaviour of Czech Generation Z. International research likewise indicates that sports communication practices can vary across cultural and organisational contexts (30). International research from other sporting environments further demonstrates the importance of social media within fan communication (30), although findings from such contexts cannot automatically be transferred to Czech sports organisations.

More recent evidence provides a stronger empirical basis for examining social media within the Czech environment. Opelík et al. (31) analysed Instagram marketing communication among four professional basketball clubs in the Czech Republic using quantitative and qualitative approaches. Their study identified differences and deficiencies in club communication and emphasised the practical importance of strategically managing Instagram content to improve fan engagement and reach. This evidence is particularly relevant because it demonstrates that platform-specific social media management is already an identifiable issue within Czech professional sport. Similarly, Voráček et al. (9) place social-network communication within the broader strategic framework of digital marketing by sports organisations. Together, these studies provide a stronger foundation for investigating how Czech sports teams use digital platforms and how audiences respond to their content.

Evidence from the wider Czech digital marketing environment also supports the importance of platform-specific analysis. Dolénkováet al. (32) examined Instagram as an influencer-marketing tool in the Czech Republic and found that different Instagram communication formats offer different possibilities for reaching audiences. Although their study concerns influencer marketing rather than sports teams, it demonstrates that the Czech social media environment has platform- and format-specific characteristics that merit empirical investigation. Such findings complement sport-specific Czech evidence and reinforce the need to avoid assuming that international findings automatically describe local patterns.

Research examining Czech social media communication outside sport has similarly found that the nature of content can influence audience response. For example, engagement analysis of social media content associated with Czech television communication highlighted the relevance of behind-the-scenes and authentic content (33). This is consistent with broader research suggesting that visually appealing and audience-relevant content may foster stronger interaction (1, 24). At the same time, the continued development of TikTok, Instagram, and other visually oriented platforms is reshaping digital marketing practices (23, 34). Algorithmic content curation and changing user practices further increase the need for organisations to continuously evaluate rather than simply assume the effectiveness of particular communication strategies (15, 16).

Taken together, the literature demonstrates the strategic relevance of social media in contemporary sport while also revealing several limitations in current knowledge. First, much of the existing research focuses on international professional sport, individual athletes, sponsorship, or broader brand outcomes, whereas comparative evidence concerning TikTok and Instagram among Czech sports teams remains limited. Second, existing Czech research has begun to examine Instagram and digital sports communication (9, 31), but direct comparative evidence across TikTok and Instagram remains scarce. Third, the literature indicates that platform characteristics, content type, and publication timing may be associated with audience interaction, but these relationships should be empirically examined rather than assumed. Finally, observable social media engagement should be distinguished from broader constructs such as brand loyalty, brand equity, or long-term marketing effectiveness.

The present study addresses these gaps by comparing observable engagement performance on TikTok and Instagram among selected Czech sports teams using Engagement Rate (ER), Love Rate (LR), and Talk Rate (TR), while additionally examining descriptive patterns associated with content type, publication day, and posting time. The study thereby extends existing Czech sport-social-media research beyond a single platform and provides evidence from sports organisations differing in size and sporting discipline. The analysis is positioned within the wider strategic role of social media in sport identified by Koronios (11) and recent sport-management research (21), while maintaining a clear distinction between observable engagement and broader brand or sponsorship outcomes.

Based on the preceding theoretical and empirical evidence, the following hypotheses were postulated;

**H1:** There is a significant difference in engagement performance between TikTok and Instagram among the selected Czech sports teams.

The alternative hypothesis reflects the possibility that differences in platform architecture, content formats, audience interaction mechanisms, and communication practices are associated with different levels of observable engagement. Previous research indicates that platform-specific characteristics and content strategies can shape audience interaction in digital sport communication (3, 9, 31). Importantly, the hypothesis concerns differences in engagement performance and does not assume in advance that either TikTok or Instagram will outperform the other.

**H0:** There is no significant difference in engagement performance between TikTok and Instagram among the selected Czech sports teams.

The null hypothesis reflects the possibility that observable engagement outcomes do not differ systematically between the two platforms and may instead be associated with content characteristics, communication practices, account-specific factors, or other contextual conditions. Previous research demonstrates that both TikTok and Instagram can facilitate audience interaction when content is adapted to platform formats and audience expectations (3, 4, 6).

## 3 Material and methods

### 3.1 Research design and study scope

This study employed a quantitative observational research design combining social media content analysis with descriptive and inferential statistical procedures. The analytical focus was engagement performance on Instagram and TikTok among selected Czech sports organisations. Engagement performance was operationalised through platform-based interaction metrics, specifically Engagement Rate (ER), Love Rate (LR), and Talk Rate (TR), together with descriptive characteristics relating to content category, publication day, and posting time. The study was designed as a comparative analysis of organic social media communication rather than as an assessment of overall brand performance. Accordingly, ER, LR, and TR were treated as indicators of observable audience interaction with published content and not as direct measures of brand loyalty, brand equity, audience attitudes, long-term brand development, or competitive positioning. This distinction was maintained throughout the analysis to ensure consistency between the research objective, the measured variables, and the interpretation of the findings. Accordingly, the analysis examined platform-level differences in engagement performance and descriptively explored variation associated with content characteristics and publication timing. The observation period extended from 1 January 2024 to 13 October 2024. Only content publicly available through the analysed organisational social media profiles was included. The study focused on communication produced and published by sports organisations and did not use individual-level information about social media users.

### 3.2 Sample and selection of social media accounts

The analytical sample comprised 15 different Czech sports teams or sports organisations representing six sports: football, ice hockey, volleyball, beach volleyball, handball, and basketball. Nine organisations were analysed through Instagram and six through TikTok. The Instagram and TikTok groups consisted of different organisational accounts; consequently, the design represents a comparison between two sets of sports accounts rather than a matched within-team comparison in which the same teams were observed simultaneously on both platforms. The sample was heterogeneous with respect to sport and social media audience size and included organisations with substantially different follower bases. The selection was purposive rather than probabilistic. The account selection was purposive and aimed to include Czech sports organisations representing different sports and audience sizes. The sample should therefore be understood as a purposively assembled set of Czech sports organisations suitable for an exploratory comparison of social media engagement patterns rather than as a statistically representative sample of the entire Czech sports sector.

The Instagram sample comprised AC Sparta Praha (football), SK Slavia Praha (football), HC Sparta Praha (ice hockey), HC Kometa Brno (ice hockey), VK Lvi Praha (volleyball), VK Prostějov (volleyball), Czech Beach Volleyball/Český volejbal—beach (beach volleyball), HC Dukla Praha (handball), and ERA Basketball Nymburk (basketball). The TikTok sample comprised FC Viktoria Plzeň (football), HC Dynamo Pardubice (ice hockey), VK Jihostroj České Budějovice (volleyball), Czech Beach Volleyball representative content (beach volleyball), Talent tým Plzeňského kraje (handball), and BK Opava (basketball). This composition provided representation of all six sports in the study while allowing the two platforms to be examined across different organisational and sporting contexts. However, because the organisations represented on Instagram and TikTok were not identical, observed platform differences may partly reflect differences between the organisations and sports accounts included in each platform group. This design characteristic was taken into account when interpreting the results and limits the extent to which differences can be attributed exclusively to the platform itself.

### 3.3 Data collection and analytical corpus

Data were collected from publicly accessible Instagram and TikTok profiles for the period from 1 January to 13 October 2024. Social media metrics were obtained using the Popsters analytics platform. The resulting analytical corpus comprised 7,721 social media content items**.** Of these, 5,254 were standard Instagram posts, 1,748 were Instagram Reels, and 719 were TikTok videos. Instagram content therefore accounted for 7,002 observations in total, whereas TikTok accounted for 719 observations. The unequal number of observations across platforms reflects the volume and format of content available from the selected accounts during the observation period. No artificial balancing or random subsampling was applied to equalise the number of Instagram and TikTok observations. Consequently, the descriptive results reflect the actual composition of the analysed corpus.

The data collection focused on content characteristics and observable interaction indicators available for the public profiles. The variables considered in the study included the platform and content format, publication frequency, day and time of publication, visual and textual characteristics relevant to content classification, and engagement indicators represented by ER, LR, and TR.

The study focused on organic content available through the selected public accounts. Paid social media advertising was not included as a separate analytical component. Importantly, the category labelled Sponsored in the content analysis did not refer to paid or boosted social media advertisements. Instead, it referred to organically published posts in which a team sponsor, commercial partner, or sponsorship-related message formed part of the content. This distinction was maintained because sponsorship-related editorial content can be present on an organisation's public profile without constituting paid platform advertising.

Because the study used publicly available organisational social media content, no private user profiles, direct messages, or non-public individual-level data were accessed or analysed.

### 3.4 Engagement measures

Engagement performance was operationalised using three social media interaction indicators: Engagement Rate (ER), Love Rate (LR), and Talk Rate (TR). The metrics were obtained and calculated through the Popsters analytics environment used for data collection.

**Engagement Rate (ER)** represented the overall level of observable interaction associated with social media content and was used as the principal indicator of audience engagement.

**Love Rate (LR)** captured engagement expressed specifically through likes and was therefore interpreted as an indicator of positive, low-effort audience interaction with published content.

**Talk Rate (TR)** captured engagement expressed through comments and was used to represent the extent to which published content generated explicit audience responses and conversation.

The three indicators were analysed separately because they represent different forms of observable social media interaction. A like requires a different form and intensity of user response than a written comment, while the broader ER indicator captures overall engagement behaviour. For this reason, LR and TR were not treated as interchangeable measures.

Most importantly, these measures were interpreted strictly as engagement metrics. They were not considered direct measures of brand loyalty, psychological attachment, brand equity, purchasing behaviour, long-term supporter retention, or other latent marketing constructs. Such outcomes would require additional behavioural, survey-based, experimental, or longitudinal evidence that was outside the scope of the present study. This operational distinction is particularly important for interpreting the findings concerning differences between Instagram and TikTok.

Engagement rate (ER) was calculated using Equation (1).

$$ER_{\mathrm{post}}=\;\frac{\left(\mathrm{Likes}+\mathrm{Shares}+\mathrm{Comments}\right)}{\mathrm{Followers}}x\;100\text{\%}$$ (1)

Love Rate (LR), which captures engagement through likes, was calculated using Equation (2).

$$\mathrm{LR}=\frac{\mathrm{Total}\;\mathrm{Likes}}{\mathrm{Follower}\;x\;\mathrm{Posts}}\;x\;100\text{\%}$$ (2)

Talk Rate (TR), which captures engagement through comments, was calculated using Equation (3).

$$\mathrm{TR}=\frac{\mathrm{Total}\;\mathrm{Commnets}}{\mathrm{Follower}\;x\;\mathrm{Posts}}\;x\;100\text{\%}$$ (3)

These metrics are widely used in social media research to evaluate engagement and interaction levels, as cited in Westerbeek (16).

### 3.5 Content classification and coding procedure

In addition to the quantitative analysis of the complete corpus of 7,721 content items, a more detailed manual content classification was conducted for a selected subset of 115 posts. The subset comprised 30 TikTok videos, 45 standard Instagram Posts, and 40 Instagram Reels. The purpose of this additional analysis was to descriptively examine content characteristics represented within the selected subset across the three analysed formats. The coding was conducted by one researcher, who was also the corresponding author of the study. Thus, the qualitative content classification represents a single-coder procedure.

An initial coding framework comprising six broad content categories was developed before the final analysis. These preliminary categories were intended to capture recurring types of sports social media communication, including informational content, team-related highlights, player-focused communication, fan-engagement initiatives, sponsorship-related content, and humorous or entertainment-oriented content. The initial conceptualisation was informed by previous research addressing the role of content characteristics in social media engagement.

During the manual examination of the selected subset, however, the initial six-category framework proved insufficient to represent the diversity of the observed posts. Additional recurring content patterns emerged from the material. The coding framework was therefore expanded inductively during the analytical process. The final set of categories corresponds to the categories reported in Table 3 and includes, among others, Player/Athlete Persona, Fan Engagement, Current Events, Match Previews, Match Highlights/Results, Trend-related content, and Sponsored content.

The final coding procedure therefore combined an initial deductive framework informed by previous research with an inductive refinement based on patterns identified in the analysed social media content. This approach allowed the coding system to capture forms of communication that had not been sufficiently represented by the original six-category framework.

Categories were not necessarily mutually exclusive. A single content item could contain characteristics of more than one category. Where a post represented multiple content categories, its contribution was divided between the relevant categories rather than being assigned exclusively to one category. This procedure explains the fractional values, such as **0.5 and 1.5**, reported in Table 3. These values therefore represent distributed category attribution and not fractional numbers of actual social media posts. Because the coding was conducted by a single researcher, inter-rater reliability statistics such as Cohen's kappa could not be calculated. This represents a methodological limitation of the study and is acknowledged when interpreting the content-analysis findings. The content-category results should consequently be understood primarily as a systematic descriptive component complementing the quantitative engagement analysis.

### 3.6 Statistical analysis

The quantitative analysis combined descriptive statistics with one-way analysis of variance (ANOVA). Statistical calculations associated with the inferential analysis were performed in Python. Descriptive statistics were first used to characterise the distribution of social media content and the observed engagement patterns across platforms, content formats, publication days, and posting times. Engagement Rate (ER), Love Rate (LR), and Talk Rate (TR) were considered separately to distinguish different dimensions of observable audience interaction.

One-way ANOVA was used as an exploratory inferential procedure to assess whether mean engagement differed across the groups examined in the available analysis. Separate ANOVA comparisons were reported for the platform-related analysis, publication day, and posting time. For each ANOVA, the null hypothesis stated that the relevant group means were equal, whereas the alternative hypothesis stated that at least one group mean differed from the others. Statistical significance was assessed at *α* = 0.05. A *p*-value below 0.05 was considered statistically significant, whereas *p* ≥ 0.05 was interpreted as insufficient evidence to reject the null hypothesis. A statistically non-significant result was therefore not interpreted as evidence that the compared groups were equivalent.

For the platform-related analysis, the ANOVA assessed whether the observed differences in engagement between the examined platform or format groups were statistically distinguishable from within-group variation. This analysis provided the inferential basis for evaluating H1 and H0 formulated above. The analyses of publication day and posting time were conducted analogously to examine whether mean engagement varied across the respective temporal groups. Where the omnibus ANOVA was non-significant, no *post-hoc* pairwise comparisons were interpreted, because the analysis did not provide evidence of an overall difference among the examined group means.

Inferential results were interpreted together with the descriptive findings rather than treating numerical differences alone as evidence of statistical superiority. Consequently, higher descriptive ER, LR, or TR values for a particular platform, format, publication day, or posting time were not interpreted as statistically confirmed differences when the corresponding inferential analysis was non-significant. LR- and TR-specific comparisons for which no separate inferential tests were available were treated as descriptive only.

The interpretation of the ANOVA results requires several methodological qualifications. The retained analytical documentation does not contain the complete ANOVA specification, including degrees of freedom, assumption diagnostics, effect sizes, and confidence intervals. It was therefore not possible to retrospectively verify assumptions such as homogeneity of variance or to quantify the magnitude and precision of the reported group differences. The reported ANOVA results are consequently treated as exploratory rather than definitive inferential evidence.

In addition, the analytical structure of the dataset does not fully satisfy the independence assumption underlying conventional one-way ANOVA. Individual social media posts were nested within a limited number of organisational accounts, meaning that observations originating from the same account may be more similar to one another than observations originating from different accounts. The numbers of observations were also substantially imbalanced between Instagram and TikTok, with 7,002 Instagram items and 719 TikTok items in the complete analytical corpus. Furthermore, the Instagram and TikTok samples consisted of different organisational accounts rather than matched observations of the same teams on both platforms. These characteristics mean that observed differences may reflect account- and organisation-specific factors in addition to platform-related variation.

A multilevel or mixed-effects modelling approach, with individual posts nested within organisational accounts, would provide a more explicit means of accounting for this hierarchical structure and separating within-account from between-account variability. Such a re-analysis could not be conducted within the available analytical framework. The ANOVA findings were therefore interpreted conservatively as exploratory evidence concerning the selected sample, and the absence of a multilevel re-analysis is recognised as a limitation of the study.

### 3.7 Treatment of the unusually high talk rate observation

During descriptive analysis of Talk Rate, the TikTok account of FC Viktoria Plzeň exhibited an unusually high TR value of 0.8368% relative to the remaining observations. As no predefined statistical outlier criterion was applied, this observation was not treated as a formally detected statistical outlier.

Instead, the calculation excluding this unusually high value was interpreted as a *post-hoc* descriptive sensitivity check**.** The purpose of this additional calculation was solely to determine the extent to which the aggregate TikTok TR was influenced by one exceptionally high observation. The primary observation was retained conceptually as part of the analysed evidence, while the recalculated value without it was reported separately for descriptive comparison.

Accordingly, no claim is made that the FC Viktoria Plzeň observation was erroneous, invalid, or statistically eligible for exclusion. The sensitivity calculation is used only to demonstrate the influence of an extreme value on the aggregated descriptive result.

### 3.8 Scope of inference and methodological limitations

The study was observational and based exclusively on publicly observable social media activity. Relationships identified between platform, content characteristics, publication timing, and engagement metrics should therefore be interpreted as associations and descriptive patterns rather than causal effects. In particular, the design does not permit causal attribution of observed differences to platform algorithms, audience characteristics, or other mechanisms that were not directly measured.

The study did not assess follower demographics, audience motivations, brand loyalty, brand equity, purchase intentions, sponsor recall, competitive positioning, or long-term brand development. Conclusions are therefore restricted to observable engagement performance and content-related interaction patterns.

The purposive sample, differences between the organisations represented on the two platforms, unequal numbers of observations, single-coder content classification, and limitations of the available post-level analytical data constrain the generalisability of the findings. The results should therefore be interpreted as evidence relating to the selected Czech sports organisations during the defined 2024 observation period rather than as estimates applicable to the wider Czech or international sports sector. These limitations are considered further in Section 4.7.

## 4 Results

### 4.1 Distribution of content and audience interactions

The analytical corpus comprised 7,721 content items published between 1 January and 13 October 2024 across nine Instagram accounts and six TikTok accounts representing the selected Czech sports organisations. The dataset included 5,254 standard Instagram posts, 1,748 Instagram Reels and 719 TikTok videos, accounting for approximately 68%, 23% and 9% of the corpus, respectively. Instagram therefore contributed 7,002 content items in total. These proportions describe the composition of the sampled content rather than platform adoption across the Czech sports sector.

Table 2 presents the distribution of posts, likes and comments. Standard Instagram posts generated 20,224,304 likes and 116,250 comments, Instagram Reels generated 9,482,379 likes and 44,901 comments, and TikTok videos generated 3,299,201 likes and 41,121 comments. Across all three groups, the recorded totals were 33,005,884 likes and 202,272 comments.

**Table 2:** Distribution of analysed content and audience interactions.

Content format	Post count	Post share	Like count	Like share	Comment count	Comment share
Instagram posts	5,254	68%	20,224,304	61%	116,250	57%
Instagram Reels	1,748	23%	9,482,379	29%	44,901	22%
TikTok videos	719	9%	3,299,201	10%	41,121	20%
**Total**	**7,721**	**100%**	**33,005,884**	**100%**	**202,272**	**99%** ^a^

Source: Researcher.

a Percentages may not sum to exactly 100% because of rounding.

The number of Instagram Reels was approximately 2.43 times the number of TikTok videos. Nevertheless, TikTok generated only 3,780 fewer comments than Instagram Reels, equivalent to approximately 8.4% fewer comments when the Reels total is used as the denominator. This comparison identifies relatively substantial commenting activity within the smaller TikTok corpus. However, aggregate interaction counts do not account for differences in follower numbers, exposure or account composition and should be distinguished from the engagement rates reported below.

### 4.2 Engagement rate across platforms and formats

The descriptive comparison identified higher Engagement Rate (ER) values for TikTok video content than for Instagram Reels, with reported values of 15.98% and 7.36%, respectively. The TikTok value was therefore more than twice the corresponding Reels value. The reported one-way ANOVA associated with the platform-related comparison returned *F* = 0.96 and *p* = 0.42, with a reported critical F value of 4.26. At the specified significance threshold of *α* = 0.05, the analysis did not identify a statistically significant difference between the examined group means. Given the limitations of the analytical specification, this result should be interpreted as exploratory and does not provide sufficient evidence to support H1. This result does not establish equivalence between platforms or demonstrate that the observed numerical differences are negligible. It indicates only that the reported analysis did not identify a statistically significant difference in mean engagement. As specified in the Methods, the ANOVA is interpreted as exploratory because the available analytical structure does not permit re-analysis accounting for posts nested within accounts.

### 4.3 Engagement rate by publication day

Variation in engagement according to publication day was examined using ANOVA. The reported ANOVA returned F = 7.113 × 10^−12^ and *p* = 1.00 and therefore did not identify statistically significant differences between the examined group means at *α* = 0.05. Given the limitations of the available analytical specification, this result should be interpreted as exploratory and does not establish equivalence between publication days or identify an optimal posting day.

### 4.4 Engagement rate by publication time

Variation in engagement according to publication time was examined using ANOVA. The reported ANOVA returned *F* = 5.26 × 10^−13^ and *p* = 1.00 and therefore did not identify statistically significant differences between the examined group means at *α* = 0.05. Given the limitations of the available analytical specification, this result should be interpreted as exploratory and does not establish equivalence between publication times or identify an optimal posting window.

### 4.5 Love rate

The original descriptive analysis reported higher Love Rate (LR) values for TikTok than for standard Instagram posts and Instagram Reels. The reported differences between the two Instagram formats were approximately 0.20 percentage points for mean LR and 3.93 percentage points for maximum LR. These comparisons are interpreted as descriptive findings because no separate LR-specific inferential test was available. The reported differences should consequently not be interpreted as statistically confirmed platform superiority.

### 4.6 Talk rate and the sensitivity check

The descriptive analysis identified an unusually high account-level Talk Rate (TR) of 0.8368% for FC Viktoria Plzeň on TikTok.

The available documentation does not establish a predefined statistical criterion for excluding this observation. It is therefore retained in the principal descriptive account, while the previously reported calculation omitting it is presented separately as a *post-hoc* sensitivity check.

In that calculation, the reported mean TikTok TR decreased to 0.14%, and the maximum among the remaining observations was 0.28%. The change illustrates the sensitivity of the reported aggregate result to an exceptionally high observation; it does not establish that the observation was erroneous or justify its exclusion from the primary evidence.

The sensitivity check is therefore interpreted as a descriptive comparison and does not establish a statistically significant difference between platforms.

### 4.7 Content categories within the selected subset

Although the complete analytical corpus contained 7,721 content items, Table 3 describes a selected subset of 115 items: 30 TikTok videos, 45 standard Instagram posts and 40 Instagram Reels. It does not present category frequencies for the complete corpus.

**Table 3:** Weighted content-category distribution within the selected selected subset of 115 content items.

Content category	TikTok, *n* = 30	Instagram posts, *n* = 45	Instagram Reels, *n* = 40	Total, *n* = 115
Attracting new fans	2	3	0	5
Current Event	1	16	3	20
Fan engagement	6	4	8	18
Fan events	1.5	0	0.5	2
Match highlights, results or previews	5	13	7	25
Other	0	0	1	1
Player other	1	4	2	7
Player × fan interaction	1	0	3	4
Player/Athlete Persona	10	4	12	26
Sponsored	0.5	0	0.5	1
Trend	2	1	3	6
**Total**	**30**	**45**	**40**	**115**

Values represent weighted category contributions within the selected subset. “Sponsored” denotes organic content featuring a sponsor or commercial partner, rather than paid platform advertising. Category labels describe content characteristics or intended communication functions; they do not establish that the stated outcome, such as attracting new fans, occurred.

Source: Researcher.

The table reports weighted category attributions. Where a content item belonged to more than one category, its contribution was divided between those categories, with a total contribution of one per item. Fractional values therefore represent shared category attribution rather than fractions of individual posts. Percentages reported below use the relevant subset total as their denominator.

Player/Athlete Persona was the largest category overall, contributing 26 of the 115 weighted units (22.6%). It was followed by Match highlights, results or previews with 25 (21.7%), Current Event with 20 (17.4%), and Fan engagement with 18 (15.7%).

The category profiles differed descriptively across formats. Player/Athlete Persona accounted for 33.3% of the TikTok subset and 30.0% of the Reels subset, compared with 8.9% of the standard Instagram post subset. Fan engagement contributed 20.0% of both video subsets and 8.9% of the standard Instagram post subset.

Conversely, Current Event accounted for 35.6% of the standard Instagram post subset, compared with 3.3% of TikTok videos and 7.5% of Instagram Reels. Match highlights, results or previews contributed 28.9% of standard Instagram posts, 16.7% of TikTok videos and 17.5% of Instagram Reels. Match-related content was therefore represented in all three groups. However, because this category combines highlights, results and previews, the table does not establish the frequency of match-footage videos specifically.

These distributions characterise the composition of the selected 115-item content subset. They do not provide category-specific average engagement rates, establish statistically significant differences between categories or indicate how frequently each category occurred across all 7,721 items.

## 5 Discussion

### 5.1 Platform comparisons and the interpretation of engagement

This study compared observable engagement on TikTok and Instagram among selected Czech sports organisations and examined descriptive patterns associated with content characteristics and publication timing. TikTok displayed higher descriptive engagement values in several comparisons, including the reported ER values for video content and the LR and TR profiles. However, the reported ANOVA did not identify a statistically significant difference. The findings therefore provide exploratory evidence of variation within the sample rather than establish the superiority of either platform.

The distinction between numerical differences and statistical inference is central to interpreting these results. The reported ANOVA did not provide sufficient evidence to support H1, but this result should not be understood as confirmation that engagement is equivalent across platforms. Neither equivalence testing nor sufficiently documented uncertainty estimates were available to support that conclusion. Furthermore, the inferential findings must be considered alongside the limitations of the original analytical design.

In particular, the Instagram and TikTok groups contained different organisational accounts. Their engagement profiles may consequently reflect differences in sports, audience sizes, organisational visibility and communication practices, as well as differences associated with platforms and formats. Comparing TikTok with Instagram Reels narrows the comparison to video formats but does not resolve the differences between participating accounts. The present design cannot isolate an independent platform effect.

The descriptive results do not establish differences in the predictability or long-term consistency of engagement between platforms. Exceptionally high TikTok values indicate that substantial interaction occurred for some observations, but they do not establish how consistently such performance could be reproduced or whether it would translate into sustained audience retention.

The distinction between engagement measurement and broader marketing outcomes is consistent with the focus on digital measurement in Kennedy et al. (24). In the present study, ER, LR and TR describe recorded interactions relative to the denominators specified in the Methods. They do not directly measure the quality of audience relationships or the commercial value of those interactions.

### 5.2 Content characteristics and audience interaction

The selected 115-item content subset provides a more differentiated descriptive account of content than the platform averages alone. Player/Athlete Persona was particularly prominent among TikTok videos and Instagram Reels, while Current Event and Match highlights, results or previews were more prominent among standard Instagram posts. Fan engagement also constituted a substantial proportion of both video subsets.

The prominence of athlete-focused material is compatible with the interest in athletes as identifiable elements of sports communication developed in the athlete-brand literature (12). Research by Su et al. (4) likewise examined athlete relationship marketing through TikTok, providing a relevant context for considering personal and informal athlete content. However, those relationships should not be treated as mechanisms demonstrated by the present data. The analysis recorded content categories and engagement indicators, rather than perceived authenticity, identification with athletes or emotional attachment.

The Fan engagement category also connects the findings to research on sports communities and digital communication. Mastromartino et al. (35) discuss sports fans and communities in the context of disruption during the COVID-19 pandemic. This offers a broader conceptual context for community-oriented communication, although it does not establish that a particular format or category produces stronger relationships in the present sample.

Czech evidence provides a particularly relevant comparison. Voráček et al. (9) examined social-network communication by Czech sports federations and highlighted both informational functions and opportunities for interaction with fans. Their findings support examining informational and participatory content as potentially complementary elements of sports communication. The current study extends this discussion by describing their different representation within the selected Instagram and TikTok content subset.

Nevertheless, Table 3 cannot establish which category is most effective. A category may be prominent within the selected subset partly because it was published more frequently overall. Without the corresponding full-corpus category frequencies and engagement distributions, its probability of producing a selected subset post cannot be calculated. The table therefore identifies themes represented within the selected examples, rather than estimate the engagement advantage of choosing one theme over another.

The combined Match highlights, results or previews category also prevents conclusions about the availability or performance of match footage specifically. Its presence across all three groups contradicts a general claim that match-related content was absent from the video subsets. Broadcasting restrictions, publication delays and editorial decisions were not measured and cannot be used to explain the observed distribution. Similarly, the small contribution of Trend content does not establish that organisations avoided trends or that trend-based posts generally performed poorly.

### 5.3 Interpretation of publication timing

The reported analyses of publication day and publication time did not identify statistically significant differences between the examined group means. Given the limitations of the available analytical specification, these exploratory results provide insufficient grounds for recommending particular publication days or times. They should not be interpreted as evidence that timing is irrelevant or that engagement is equivalent across temporal groups.

Publication timing must also be distinguished from the timing of exposure and interaction. Content may accumulate engagement after its initial publication, while match schedules, content characteristics, account composition and unequal publication volumes may influence aggregated patterns. These factors were not jointly evaluated in the reported analyses.

General descriptions of digital audiences, such as Kemp (1), provide contextual information but cannot identify the demographic composition or daily routines of users interacting with the sampled accounts. The present findings therefore do not support explanations based on audience age, school or work commitments, or leisure routines.

Similarly, recommendation and distribution systems were not directly observed, and their contribution to content exposure was not measured. The study consequently cannot attribute temporal engagement patterns to platform algorithms. More detailed prospective data would be required to investigate these relationships.

### 5.4 Influential observations and the meaning of interaction metrics

The reported sensitivity calculation suggests that aggregate TikTok TR was substantially influenced by the exceptionally high FC Viktoria Plzeň observation.

Reporting the original observation alongside the sensitivity calculation makes this influence visible without treating unusual success as grounds for exclusion. In the absence of a predefined criterion, the recalculation should remain supplementary and descriptive.

The reduction in TikTok TR after omission also cautions against using a platform average as a prediction for an individual organisation. Accounts operating on the same platform may experience substantially different outcomes. The present results do not identify whether the unusually high value reflected content characteristics, audience composition, a particular sporting event or another factor.

More comments should also not automatically be interpreted as deeper or more favourable engagement. Comment totals do not distinguish supportive responses from criticism, repeated contributions or other forms of interaction. Without analysis of comment content, sentiment and unique participants, TR measures commenting activity rather than the quality of conversation. Likewise, likes provide a record of a platform action rather than a validated measure of loyalty.

These distinctions preserve the connection with the manuscript's wider sports-marketing framework while respecting the limits of its measurements. Social media has a broader strategic role within sport (11), but demonstrating that role empirically requires measures appropriate to the outcome under investigation. For example, Koronios et al. (21) examined sponsorship-related constructs and spectator engagement using survey data and structural equation modelling. Their research provides a relevant theoretical comparison, but its sponsorship findings cannot be inferred from the post-level interaction counts examined here.

### 5.5 Practical implications for Czech sports organisations

The findings offer starting points for evaluating organic communication within individual organisations. Athlete-focused and fan-oriented videos merit consideration because they were prominent in the selected TikTok and Reels content subsets. Current-event and match-related posts also warrant attention because of their substantial representation among selected standard Instagram posts. These observations can inform content trials, provided that organisations evaluate subsequent performance against their own objectives and account-level evidence.

This approach is consistent with Opelík et al. (31), whose analysis of four Czech professional basketball clubs identified differences in Instagram communication and areas for improvement. Their findings reinforce the value of examining organisational practices rather than assuming that the same approach will produce comparable results across clubs.

Publication schedules should be evaluated using each organisation's own account-level evidence. The present study does not establish optimal publication days or times. Prospective comparisons that record content characteristics, sporting context, audience size and engagement over consistent follow-up periods would provide a stronger basis for scheduling decisions.

For organisations with limited resources, the organic-content focus makes the findings relevant to editorial planning. However, production costs, staff time, advertising expenditure and conversions were not measured. The analysis therefore cannot demonstrate cost savings, reduced cost-per-action or improved return on investment. It also cannot establish that smaller organisations obtain greater benefits from TikTok than larger organisations.

Finally, the lower number of TikTok accounts and posts in this sample does not demonstrate underutilisation. Such a conclusion would require an explicit benchmark incorporating organisational objectives, adoption across eligible teams, available resources and opportunities for additional use. The present study reports publication volume among selected accounts and does not test a hypothesis about platform underutilisation.

### 5.6 Czech and wider central European relevance

The study contributes to an existing body of Czech research on sports communication rather than introduce an entirely unexplored field. Its comparative focus complements work on Czech sports federations and professional basketball clubs (9, 31). Its contribution lies in bringing together descriptive evidence on TikTok, standard Instagram posts and Instagram Reels across the selected sporting contexts.

Research outside sport also provides useful local context. Dolénková et al. (32) examined Instagram influencer communication in the Czech Republic and considered differences between available communication formats. Although their population and objectives differ from those of the present study, their work reinforces the relevance of examining formats within a specified market. It does not establish that influencer-marketing findings transfer directly to sports organisations.

The findings may inform research questions in other Central and Eastern European sports environments, particularly where organisations face comparable communication demands or resource constraints. However, these similarities must be established empirically. Geographical proximity does not demonstrate equivalent audiences, sporting structures, media arrangements or platform practices.

Accordingly, the transferable contribution is primarily an analytical focus: distinguishing platforms and formats, considering content characteristics, examining account-level variation and separating interaction metrics from wider marketing outcomes. The descriptive platform comparisons and content-category distributions require replication before they can support regional generalisations, while the reported temporal analyses remain exploratory and independently unverified.

### 5.7 Limitations and directions for future research

Several limitations constrain interpretation. First, the purposive sample included a limited number of accounts across six sports, with unequal numbers of accounts and content items across platforms. It was not statistically representative of Czech sports organisations. Moreover, the platform groups comprised different accounts, preventing a matched comparison and leaving platform differences entangled with organisational and sporting differences. The large number of posts does not compensate for the small number of organisational units.

Second, the inferential analysis did not explicitly account for posts nested within accounts. The retained documentation also does not provide the complete ANOVA specification, degrees of freedom, assumption diagnostics, effect sizes or confidence intervals required for a fully assessable inferential analysis. The extremely small reported temporal F statistics and *p*-values rounded to 1.00 cannot be independently checked against the original observations. These results are therefore retained as reported exploratory outputs and should not be treated as decisive evidence that timing is unimportant.

The post-level dataset was not available for additional re-analysis. Consequently, account clustering, alternative model specifications, uncertainty estimates, and sensitivity to analytical choices could not be further evaluated. This limitation should be a substantive limitation of reproducibility and statistical assessment. Future research should retain the analytical dataset and code and use models appropriate to the outcome distribution and hierarchical structure, with sufficient accounts to support estimation.

Third, publicly accessible data provide only a partial account of exposure and interaction. Internal measures such as reach, impressions and detailed viewing behaviour were unavailable. Follower-based rates cannot establish what proportion of exposed users interacted, and incomplete documentation of follower-count timing limits assessment of denominator comparability. Future studies should record follower counts over time, extraction dates and standardised observation periods for engagement accumulation.

Fourth, content classification was undertaken by one researcher, and inter-coder reliability was not assessed. The expansion of the coding framework and fractional allocation across categories require a reproducible codebook with definitions and decision rules. The category results available in Table 3 concern only the 115-item selected subset of 115 content items. The retained documentation does not sufficiently specify the ranking criterion and selection procedure for that subset. Its composition should therefore not be treated as representative of all posts or as evidence of category-level performance differences.

Fifth, the observation period covered part of 2024 and may not capture complete sporting seasons or longer-term changes. Competitive schedules, major events and changes in communication practices could affect the recorded patterns. The study did not isolate these influences or assess whether the observed profiles persisted subsequently.

Finally, the design did not measure audience demographics, motivations, comment sentiment, brand attitudes, purchasing behaviour, sponsorship outcomes or long-term supporter retention. Nor did it evaluate platform algorithms, broadcasting constraints or communication costs. Future work combining matched cross-platform observations with organisational interviews, internal analytics and appropriate audience measures could investigate these relationships directly. Such evidence would permit a clearer assessment of when observable engagement contributes to broader organisational objectives.

## 6 Conclusion

This study compared engagement performance on TikTok and Instagram among selected Czech sports teams and examined platform-, content-, day-, and time-specific patterns of audience interaction. TikTok displayed higher descriptive values for several engagement indicators, including ER, LR, and TR; however, the reported inferential analysis did not identify a statistically significant difference in engagement performance. Similarly, the analyses of publication day and posting time did not identify statistically significant differences. The findings therefore do not establish the superiority of either platform or support the identification of an optimal publication day or time. Rather, they demonstrate the importance of distinguishing descriptive variation in engagement from statistically confirmed differences and from broader marketing outcomes that were not measured in this study.

Analysis of the selected selected subset content subset revealed distinct descriptive content profiles across formats. Player/Athlete Persona and Fan Engagement were prominently represented among TikTok videos and Instagram Reels, whereas Current Event and Match Highlights, Results or Previews were more strongly represented among standard Instagram posts. These patterns characterise the composition of the selected selected subset content and should not be interpreted as evidence that particular content categories are inherently more effective. Nevertheless, they provide a useful empirical basis for further account-level content experimentation and evaluation by sports organisations.

Overall, the findings support a differentiated approach to organic social media communication in which platform, format, content characteristics, and organisational context are considered jointly rather than assuming a universally superior platform or content strategy. Given the observational design, purposive sample, unmatched platform groups, and limitations of the available analytical data, the findings should be interpreted as exploratory evidence from the selected Czech sports organisations rather than generalised to the wider sports sector. Future research using matched cross-platform samples, retained post-level data, and analytical methods accounting for the hierarchical structure of social media observations would provide a stronger basis for evaluating platform- and content-related differences in engagement.

## Data Availability

The original contributions presented in the study are included in the article/Supplementary Material, further inquiries can be directed to the corresponding author/s.
