# Supplementary material for: From posts to engagement: comparing TikTok and Instagram communication among Czech sports teams
Source: Front Sports Act Living. 2026 Oct 8;8:1964766. doi: 10.3389/fspor.2026.1964766 (PMC13687316; doi:10.3389/fspor.2026.1964766)
Supplement: Supplementary file 1 [file Datasheet1.pdf]

Supplementary materials
for

"From Posts to Power: Leveraging TikTok and Instagram for Strategic Brand Development of Sports Teams in the Czech Republic"

HYPOTHESES

This study has two main hypotheses;

H1: TikTok is underutilized and performs better than Instagram for sports teams in the Czech Republic.
H0: (Null Hypothesis): There is no significant difference in the effectiveness of TikTok and Instagram for sports teams in the Czech Republic.

Research Objectives

Primary Objective:

- ✓ To evaluate the effectiveness of TikTok and Instagram in sports teams brand building efforts in the Czech Republic.

SecondaryObjective1:

- ✓ ToevaluatewhichofInstagramReels,InstagramPostsandTikTokisthemosteffective basedonthefollowingmetrics:EngagementRate,LoveRate,andTalkRate(Theseare further explained in the methodology).

SecondaryObjective2:

- ✓ Determine which types of content prove most effective on each platform and guide teams on their focus areas.

LITERATURE REVIEW

Table 1: Summary of core tools used for multiple platforms

Tool/Feature	TikTok	Instagram	Description/Purpose
**Short-Form Videos**	Core Feature (15-60 sec)	Reels(upto90 sec)	Short, visually dynamic videos that are central to both platforms. Both TikTok and Instagram reels allow "carousel" posts with music to be displayed as short-form video with a swipe feature.
**LiveStreams**	Available (LiveTikTok)	Available(IG Live)	Real-time video streaming for Q & As, behind-the-scenes access, and events.
**Posts**	+Not Available	Core Feature	Posts on Instagram now supportupto20images,and videos up to 60 min long. Long-term content that stays on users' profile.
**Stories**	Available(24-hour content)	Available(24-hour content)	24-hourverticalcontentused for quick updates, polls, and behind-the-scenes.
**ForYouPage (FYP)**	Core Feature	+Not Available	TikTok's discovery-first page where users see trending and suggested content.
**Discovery/Explore Page**	+Limited(FYP serves this)	Available (Explore Tab)	Instagram's discovery page where users can explore new content from non-followed accounts.
**ARFilters/Effects**	Available	Available	Augmented realityeffectsthat overlay graphics on top of videos and photos.
**Duets /Remix**	Duets(Core Feature)	Remix(for Reels)	Users can"duet"TikToksor "remix" Instagram Reels, reacting to or collaborating with other content.

METHODOLOGY

Engagement Metrics Explanation

EngagementRate(ER)describeshowmuchfollowersengageinposts;itisseparatedforER post and ER Day:
ERpost=(likes+shares+comments)/countoffollowers*100%

ERday=(totallikes +totalshares+totalcommentsfortheday)/countoffollowers *100%

Love Rate (LR) describes how attractive each post is.There are similarities to ER, however, the focus is solely on likes. It is calculated in the following way:
LR=(totallikes)/followers/countofpostsfortheanalyzedperiod*100%

Lastly,the**TalkRate(TR)** measureshowoftenpeoplearepromotedtocommentonapost,it is a measure of how the audience communicates.
TR=(totalcomments)/followers/countofpostsfortheanalyzedperiod*100%

RESULTS

Table 2: Posts analysis.

Name	Post Count	Post %	Like Count	Like %	Comment Count	Comment %
**Post**	5 254	68%	20 224 304	61%	116 250	57%
**IG Reel**	1 748	23%	9 482 379	29%	44 901	22%
**TikTok**	719	9%	3 299 201	10%	41 121	20%
**Total**	**7 721**		**33 005 884**		**202 272**	

Source: Researcher

Figure 1

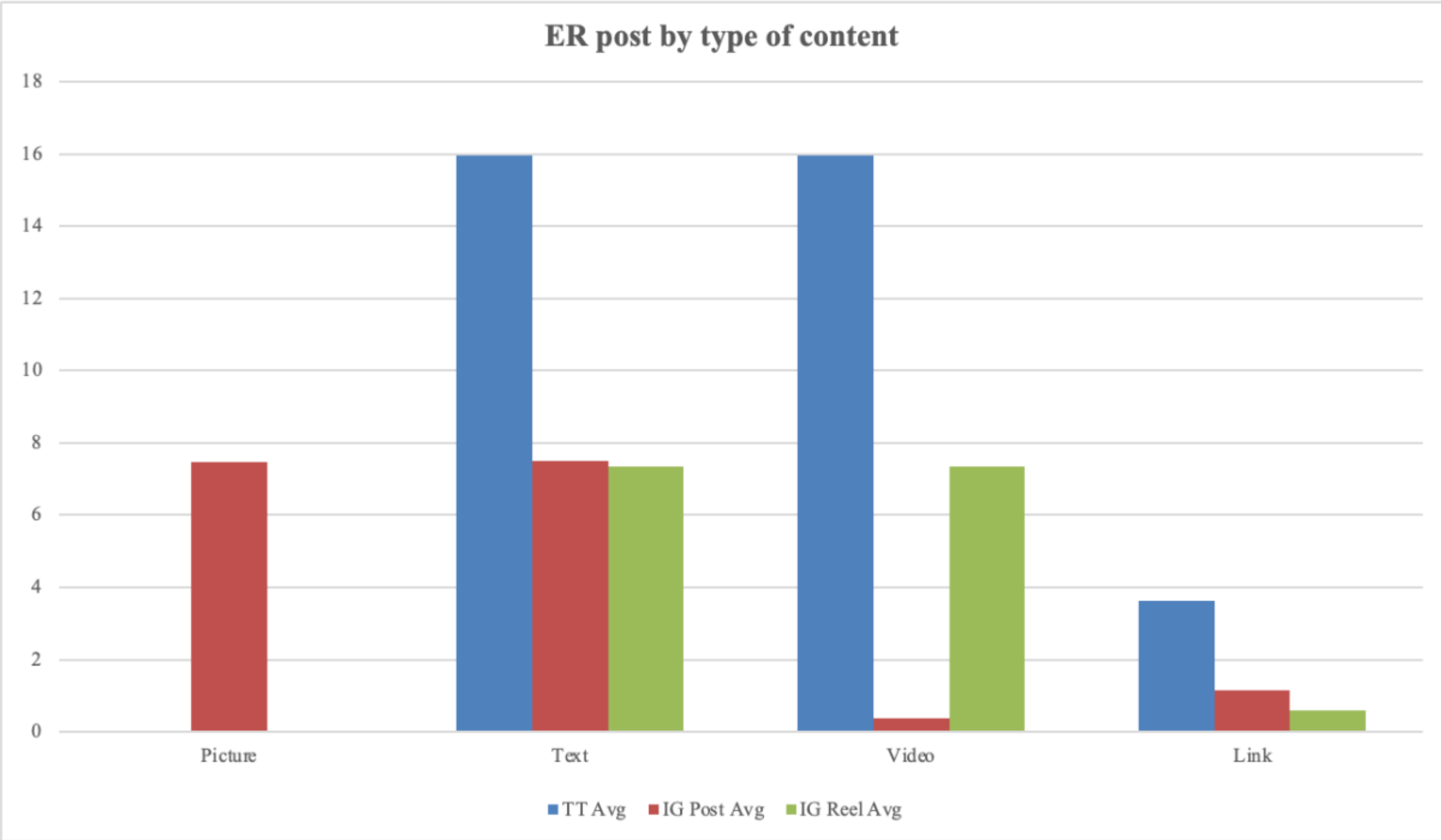

ER post by type of content
18
16
14
12
10
8
6
4
2
0
Picture
Text
Video
Link
TT Avg
IG Post Avg
IG Reel Avg

Figure 2

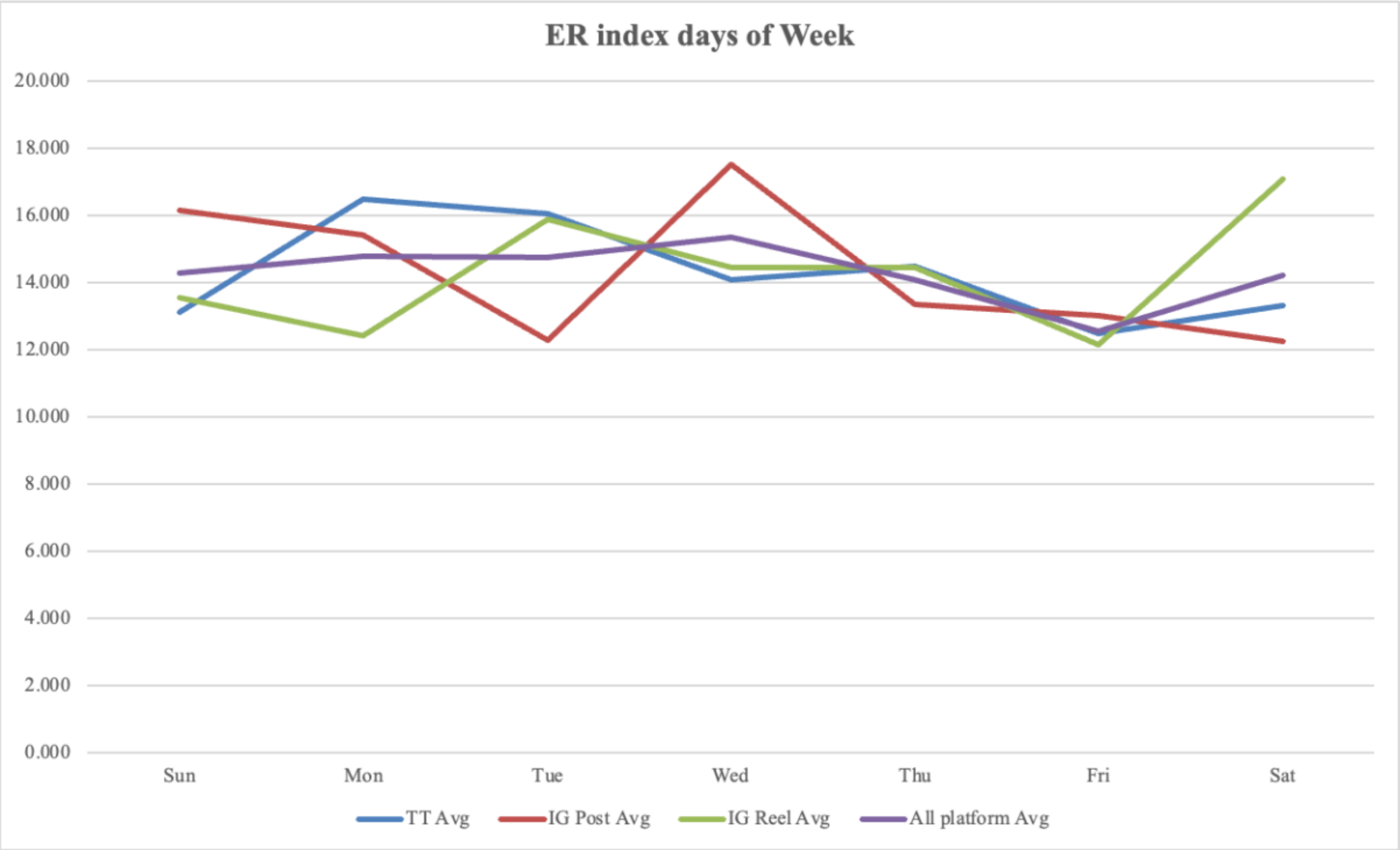
ER index days of Week
20.000
18.000
16.000
14.000
12.000
10.000
8.000
6.000
4.000
2.000
0.000
Sun
Mon
Tue
Wed
Thu
Fri
Sat
TT Avg
IG Post Avg
IG Reel Avg
All platform Avg

Figure 3

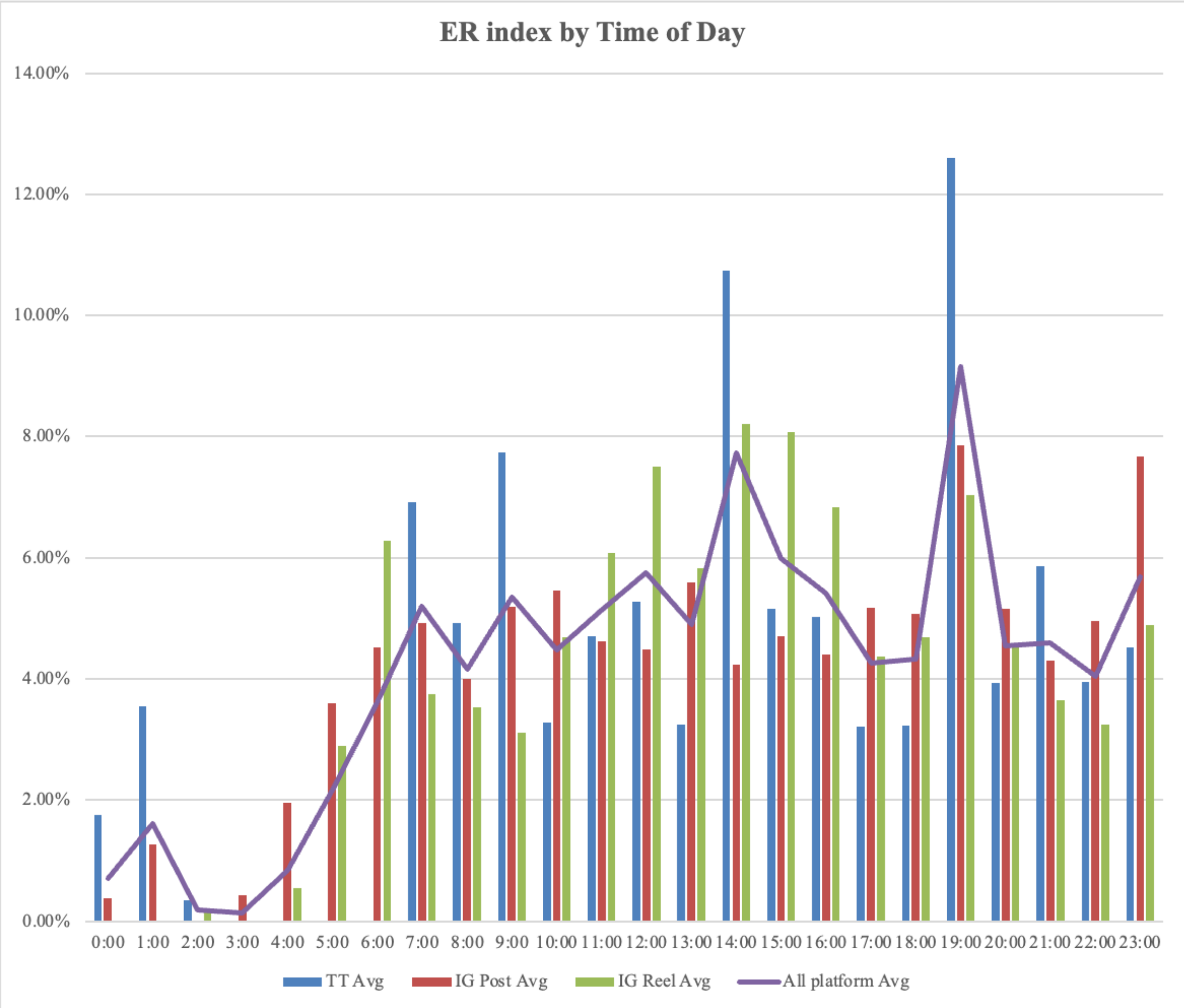

ER index by Time of Day
14.00%
12.00%
10.00%
8.00%
6.00%
4.00%
2.00%
0.00%
0:00
1:00
2:00
3:00
4:00
5:00
6:00
7:00
8:00
9:00
10:00
11:00
12:00
13:00
14:00
15:00
16:00
17:00
18:00
19:00
20:00
21:00
22:00
23:00
TT Avg
IG Post Avg
IG Reel Avg
All platform Avg

Figure 4

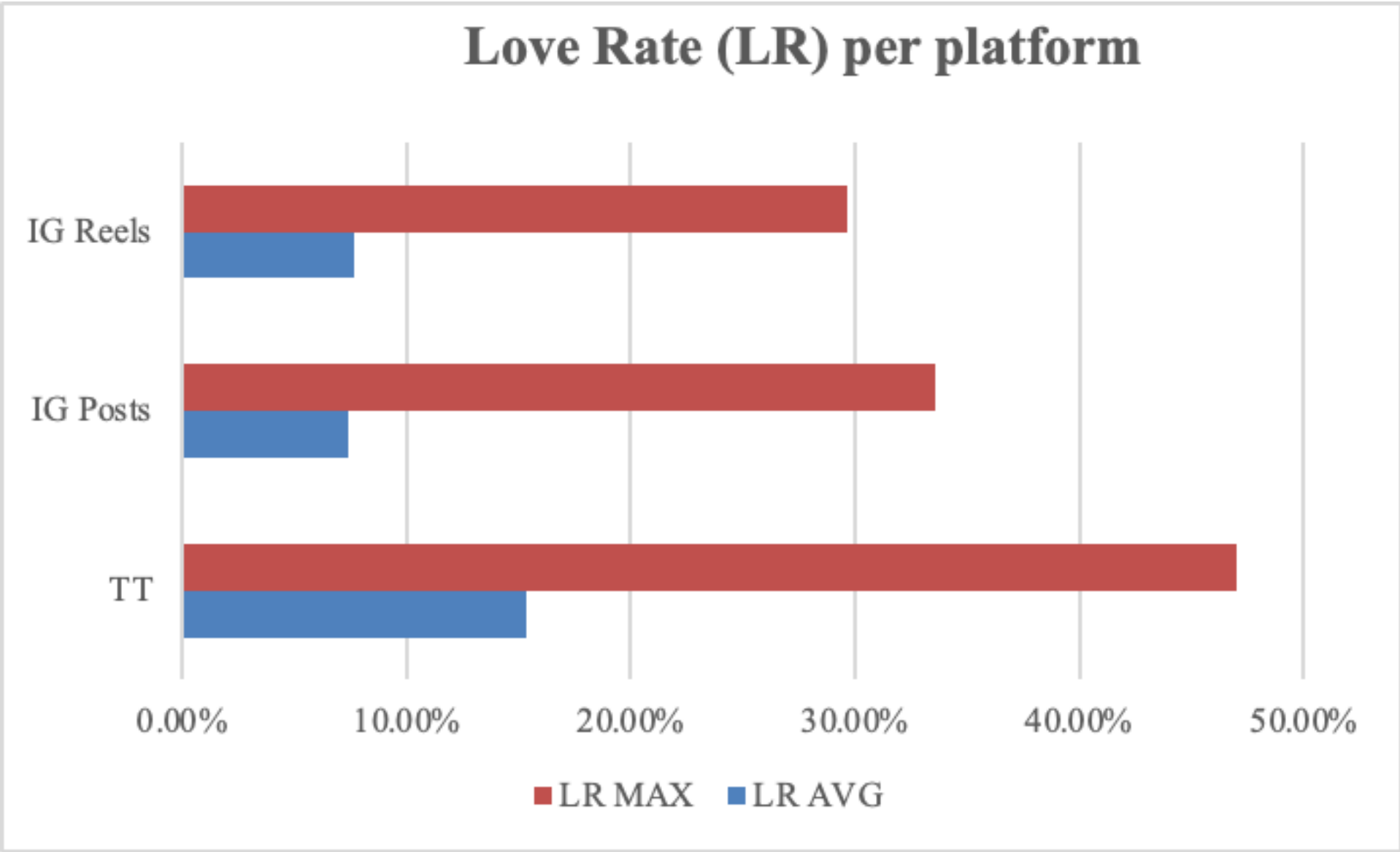
Love Rate (LR) per platform
IG Reels
IG Posts
TT
0.00%
10.00%
20.00%
30.00%
40.00%
50.00%
LR MAX
LR AVG

Figure 5

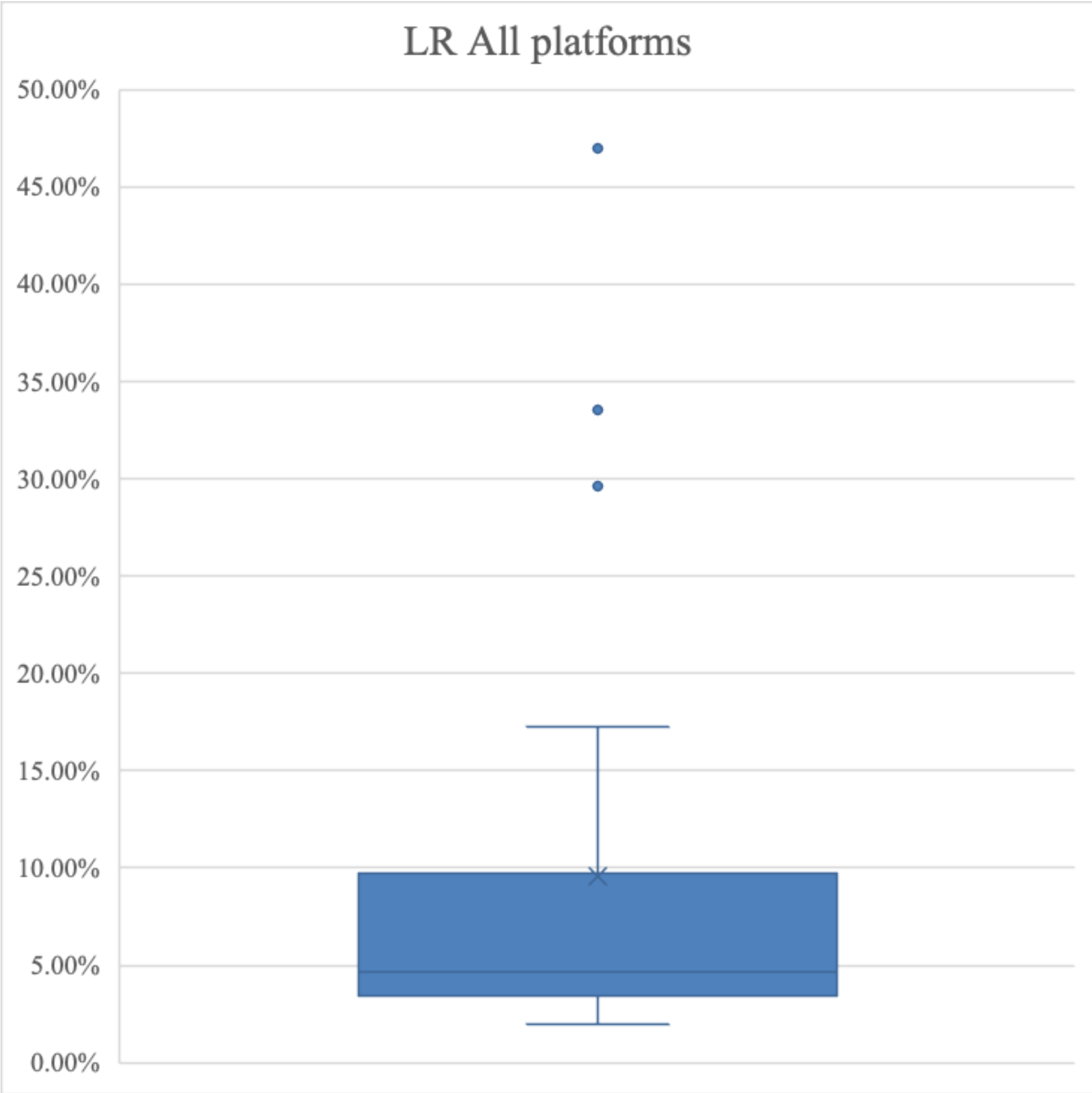

LR All platforms
50.00%
45.00%
40.00%
35.00%
30.00%
25.00%
20.00%
15.00%
10.00%
5.00%
0.00%

Figure 6

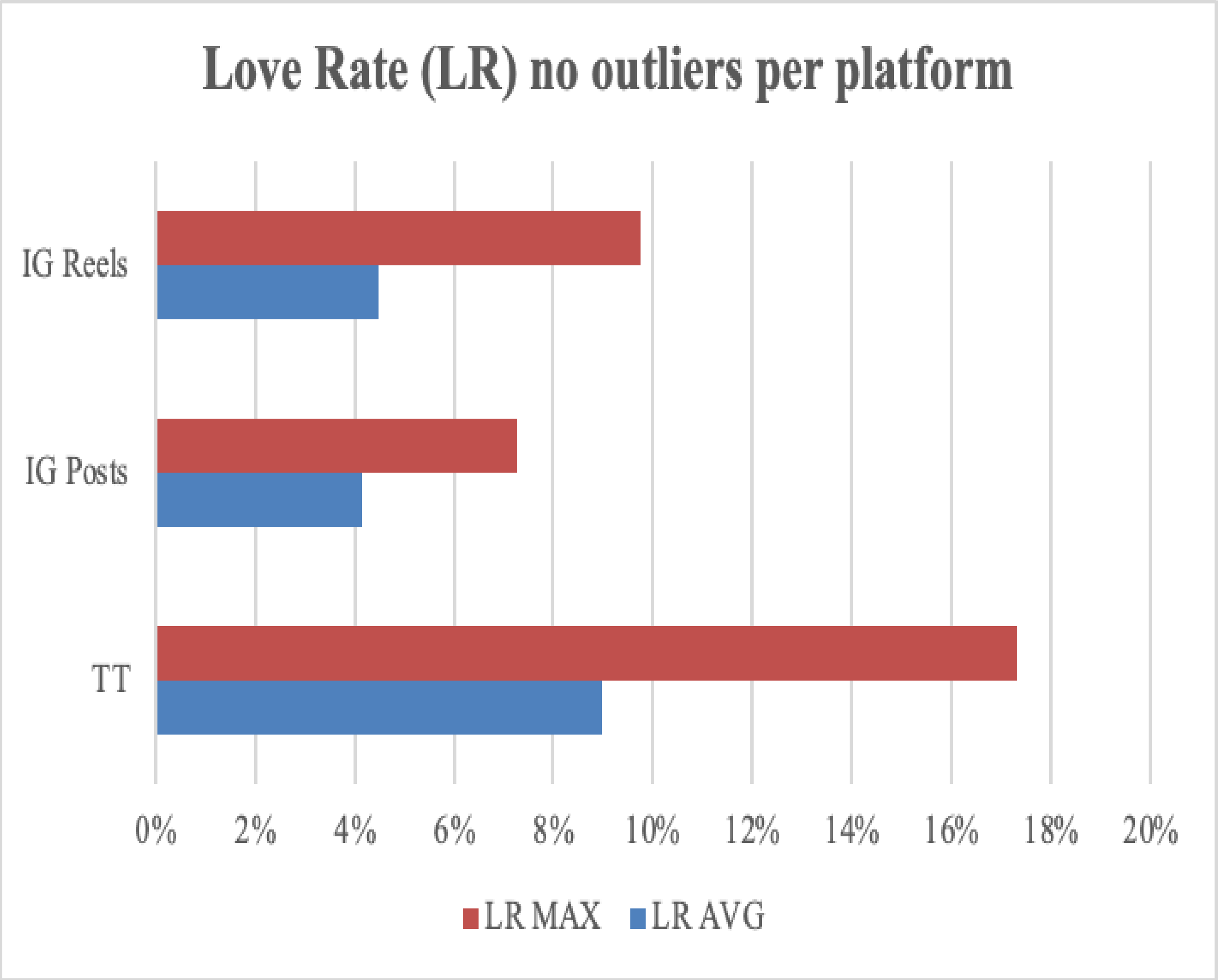

Love Rate (LR) no outliers per platform
IG Reels
IG Posts
TT
0%
2%
4%
6%
8%
10%
12%
14%
16%
18%
20%
LR MAX
LR AVG

Figure 7

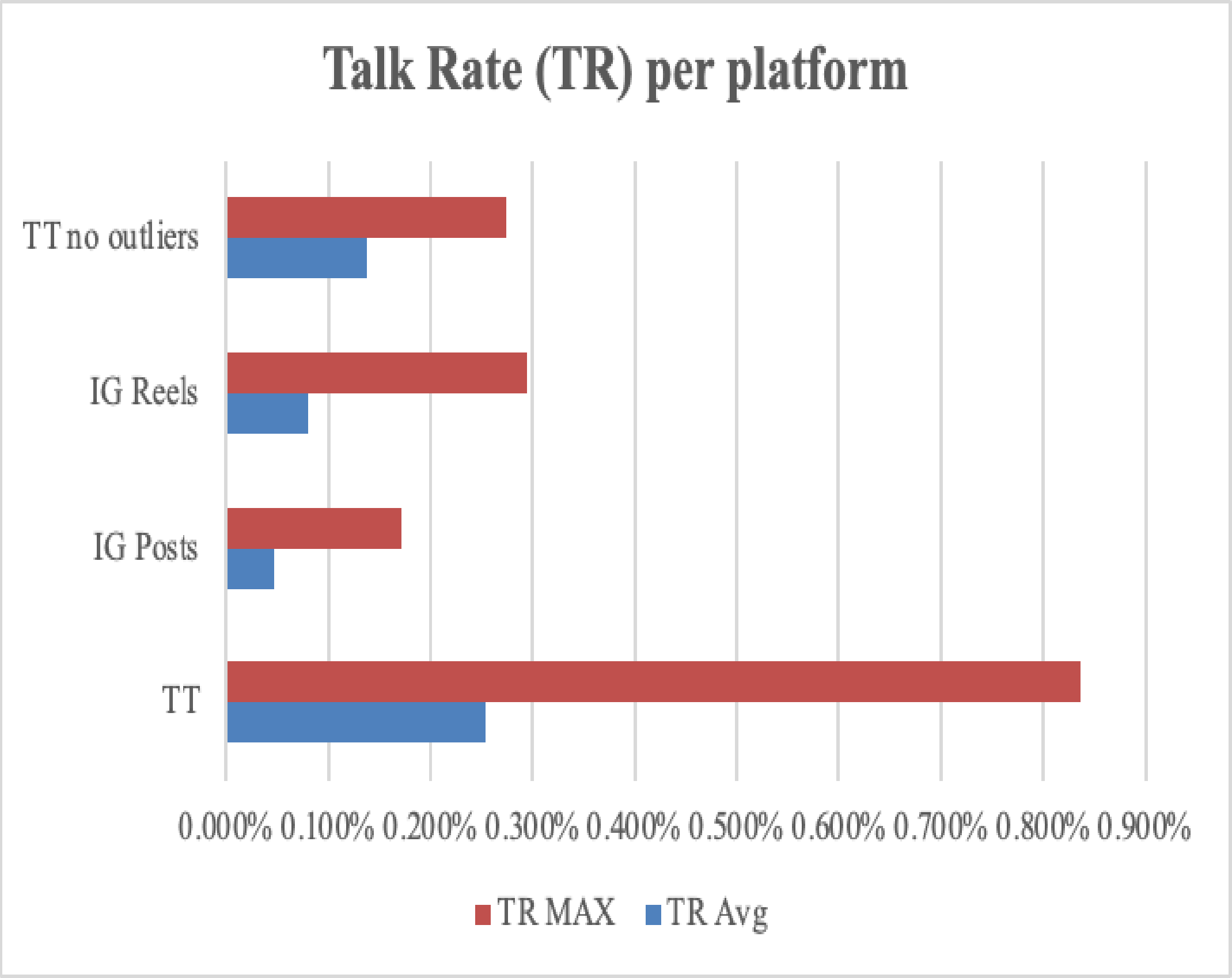
Talk Rate (TR) per platform
TT no outliers
IG Reels
IG Posts
TT
0.000% 0.100% 0.200% 0.300% 0.400% 0.500% 0.600% 0.700% 0.800% 0.900%
TR MAX
TR Avg

Figure 8

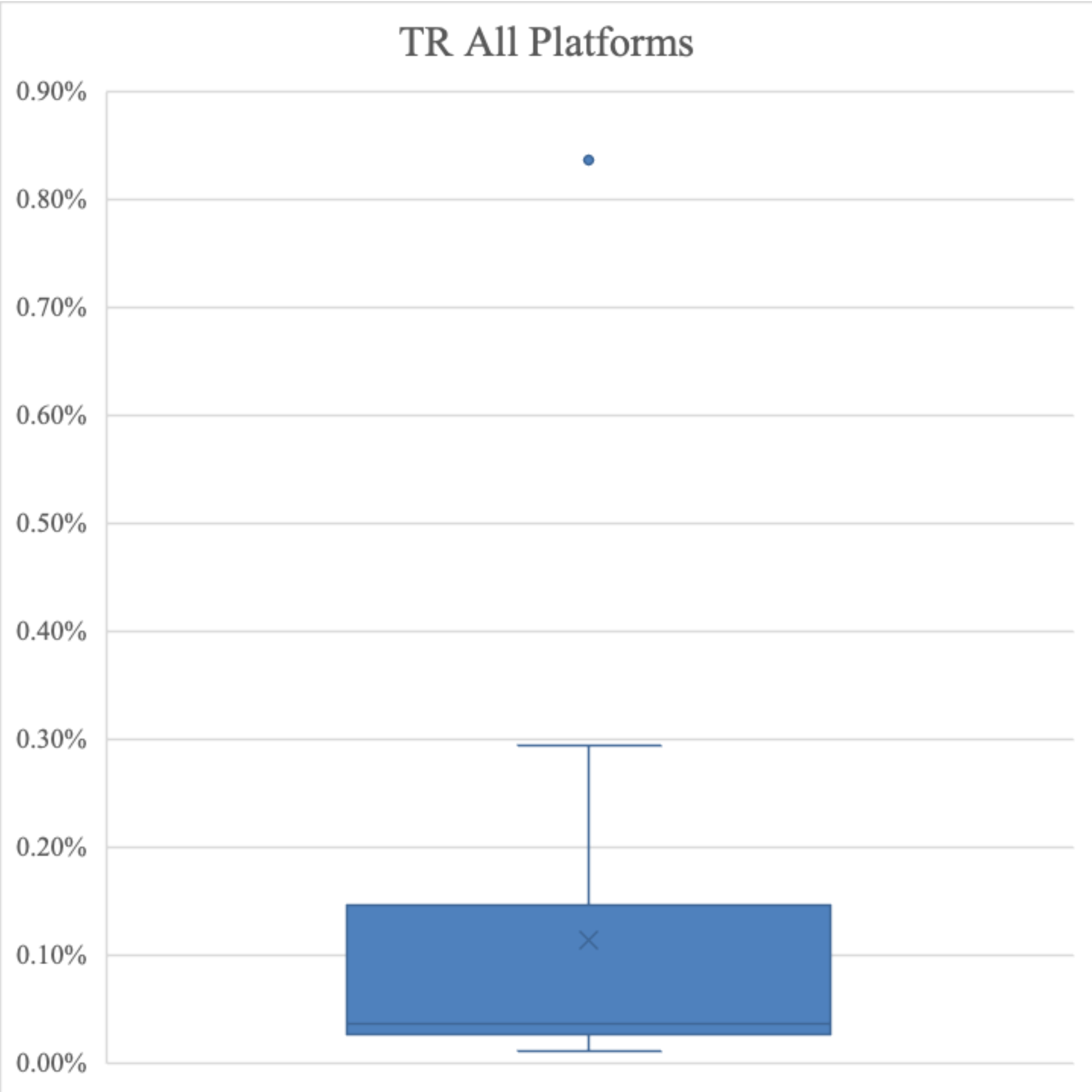

Table 3: Posts analysis

Name	TikTok	IG Posts	IG Reels	Totals
Attracting new fans	2	3	0	**5**
Current event	1	16	3	**20**
Fan engagement	6	4	8	**18**
Fan events	1.5	0	0.5	**2**
Match highlights, results or previews	5	13	7	**25**
Other	0	0	1	**1**
Player other	1	4	2	**7**
Player x fan interaction	1	0	3	**4**
Player/Athlete persona	10	4	12	**26**
Sponsored	0.5	0	0.5	**1**
Trend	2	1	3	**6**
**Total**	**30**	**45**	**40**	

Source: Researcher

Figure 9

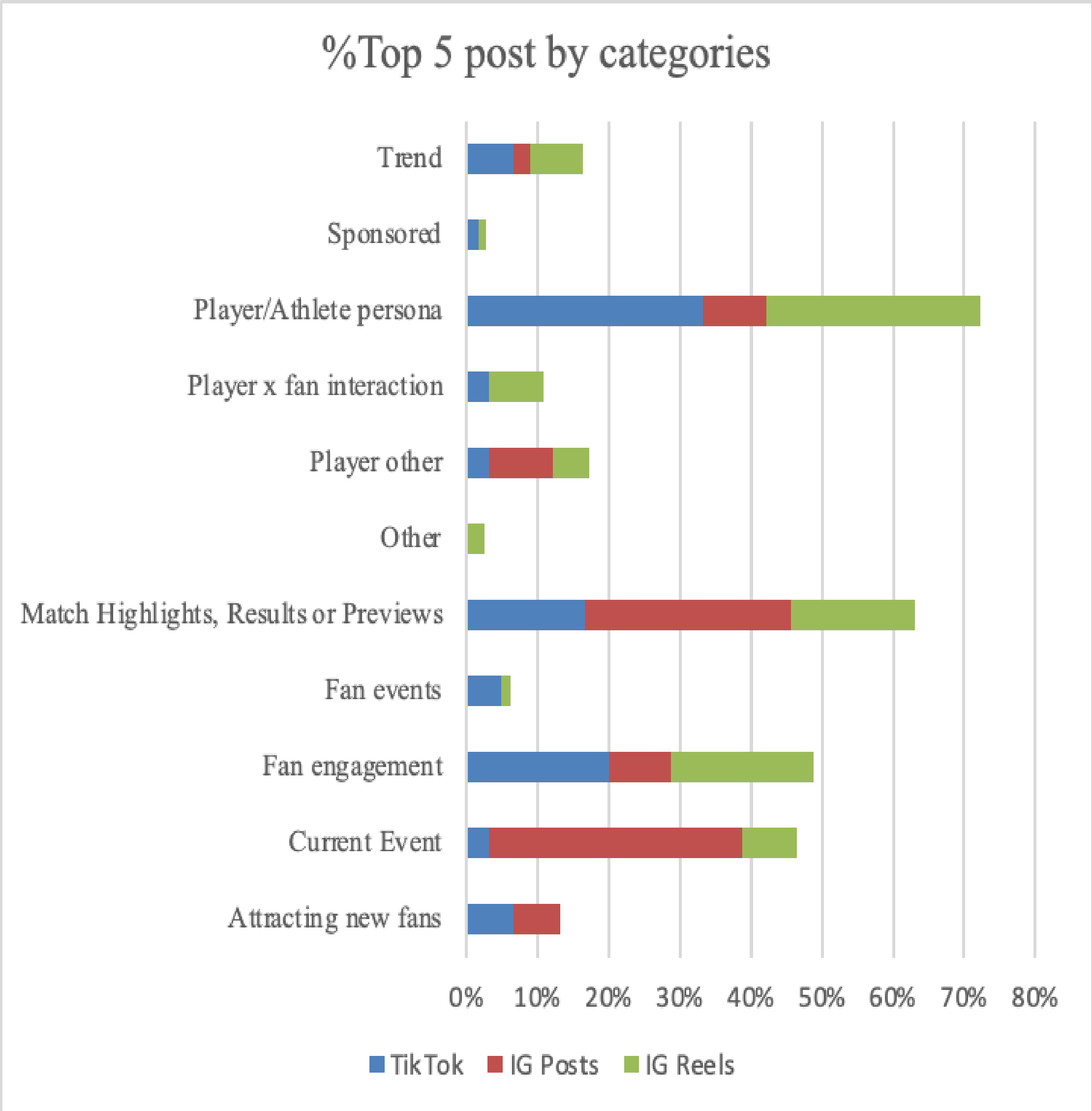

%Top 5 post by categories
Trend
Sponsored
Player/Athlete persona
Player x fan interaction
Player other
Other
Match Highlights, Results or Previews
Fan events
Fan engagement
Current Event
Attracting new fans
0%
10%
20%
30%
40%
50%
60%
70%
80%
TikTok
IG Posts
IG Reels

Figure10

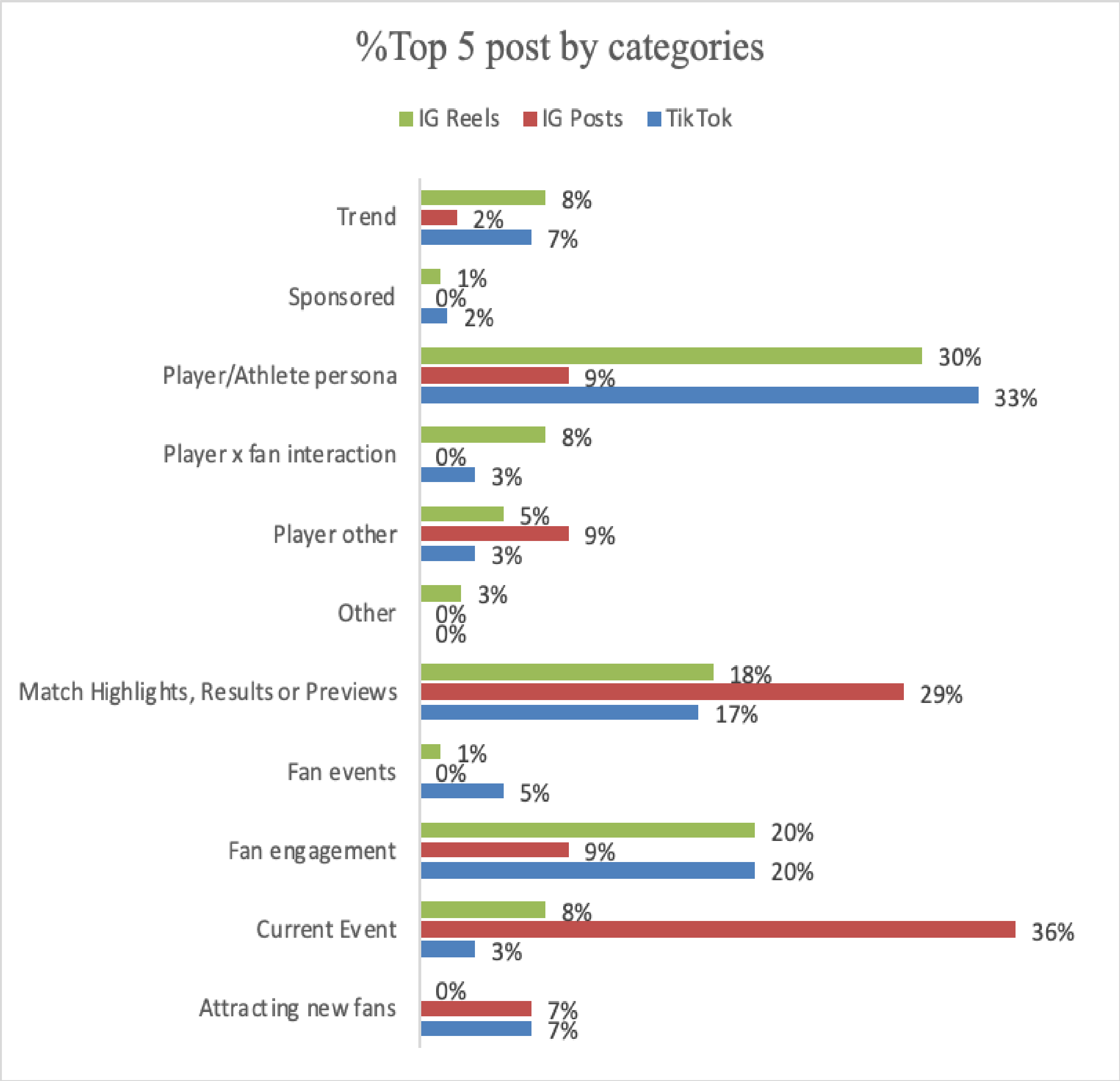

%Top 5 post by categories
IG Reels
IG Posts
TikTok
Trend
8%
2%
7%
Sponsored
1%
0%
2%
Player/Athlete persona
30%
9%
33%
Player x fan interaction
8%
0%
3%
Player other
5%
9%
3%
Other
3%
0%
0%
Match Highlights, Results or Previews
18%
29%
17%
Fan events
1%
0%
5%
Fan engagement
20%
9%
20%
Current Event
8%
36%
3%
Attracting new fans
0%
7%
7%

Conclusion

Table 4: Summary of social media results SWOT analysis

Strengths	Weaknesses	Opportunities	Threats
**Use each platform's Strength**: Ten gages younger fans with trend-driven content, IG Reels has a similar audience with less focus on trends. IG Posts are effective for sharing key info.	**Over-reliance on.** **Algorithm: Algorithm changes can drastically reduce content visibility.**	**Expansion of TikTok in the Czech Republic**: Growing TikTok adoption provides access to a wider, younger audience.	**Platform restrictions and bans**: Possible bans or regulatory restrictions on TikTok (as seen in India and the U.S.A) could disrupt strategies.
**Emotional connection with fans**: Behind-the-scenes (BTS) content builds brand loyalty and strengthens the fan-team relationship.	**Resource constraints**: Small teams lack time, budget, and expertise for consistent content production.	**Emerging AR, VR, and AI tools**: Use of AR filters (on Instagram) and AI- generated content could create immersive fan experiences.	**Algorithm changes**: TikTok and Instagram algorithms are unpredictable, which could negatively impact content visibility.
**Strong emotional appeal of Sports:** Content where strong emotions or attachment to club is displayed has a stronger impact on fans.	**Inconsistent content strategy**: Many teams lack a clear, long-term strategy, leading to ad-hoc posting.	**Live streaming for fan engagement**: Platforms prioritize live streams, giving sports teams a chance to boost real-time engagement.	**GDPR and privacy regulations**: GDPR could impose stricter rules on tracking engagement data, making it harder to measure ER, LR, and TR.
**Player-driven content**: Content where Players are displayed for their personality in general performs very well.	**Limited data** **Analytics capabilities**: Teams often lack the expertise to fully utilize analytics tools such as TikTok Analytics, and Instagram Insights.	**Collaborations with influencers and athletes**: Partnering with micro-influencers and athletes could increase brand reach and visibility.	**Emergence of new platforms**: New platforms like Threads or Lemon 8, may attract fans away from TikTok and Instagram.
**Low cost of content production**: SM are to promote your club for free or for a low price, instead of traditional platforms like billboards, tv-ads.etc.	**Unclear ROI on** **Social media**: Teams struggle to quantify how social media impacts ticket sales, sponsorships, or merchandise sales.	**Demand for BTS content**: Fans want authentic, raw, behind- the-scenes (BTS) content that builds emotional connections.	**Competition from other sports teams**: Other sports teams and leagues compete for limited fan attention, making it harder to stand out.

10 Tips for Social Media Teams to Build Their Sports Brand

1. Know Your Audience

- ✓ Develop a deep understanding of the demographics, preferences, and behaviors of your fan base on TikTok and Instagram.
- ✓ Tailor content to resonate with Czech cultural values, sports traditions, and local expressions, as this can enhance relatability and engagement.

2. Consistency Is Key

- ✓ Maintain a consistent posting schedule to sustain fan interest and improve visibility within platform algorithms.
- ✓ Ensure that all content aligns with your team's brand identity, including logos, colors, and tone of voice, to reinforce brand recognition.

3. Leverage Platform Strengths

- ✓ Take advantage of TikTok's trend-driven and viral nature by creating dynamic, entertaining, and engaging content.
- ✓ Use Instagram's Reels, Stories, and visually rich posts to support storytelling and showcase curated brand moments.

4. Engage, Don't Just Post

- ✓ Transform passive followers into active participants by hosting Q&A sessions, responding to comments, and featuring fan-generated content.
- ✓ Meaningful engagement helps build loyalty and trust while increasing visibility through platform algorithms.

5. Focus on Quality Over Quantity

- ✓ Prioritize the creation of high-quality, visually appealing content rather than posting excessively.
- ✓ On TikTok, authenticity often performs better than perfection, so embrace spontaneity when it aligns with your brand image.

6. Use Data to Drive Decisions

- ✓ Monitor engagement metrics such as Engagement Rate (ER), Like Rate (LR), and Talk Rate (TR) to determine what content performs best.
- ✓ Use data-driven insights to refine posting schedules, content themes, and content formats to maximize engagement.

7. Incorporate Local and Cultural Relevance

- ✓ Integrate Czech cultural elements, local sports traditions, holidays, and community events into your content strategy.
- ✓ Highlight local athletes and fan communities to strengthen emotional connections and increase regional support.

8. Embrace Trends but Stay Authentic

- ✓ Participate in viral trends and challenges when they are consistent with your team's brand identity.
- ✓ Avoid forced participation in trends, as it may reduce authenticity and weaken fan trust.

9. Showcase Behind-the-Scenes Content

- ✓ Give fans a glimpse into the human side of the team through behind-the-scenes videos, live streams, and exclusive content.
- ✓ Feature training sessions, player routines, and off-the-field moments to foster deeper fan connections.

10. Collaborate for Greater Reach

- ✓ Partner with athletes, influencers, and other sports teams to create co-branded campaigns and expand audience reach.
- ✓ Strategic collaborations can increase visibility, attract new followers, and strengthen brand awareness.
